# An exploratory survey of barriers and enablers of AI adoption in rural and urban dental practices in Mecklenburg-Western Pomerania, Germany

**DOI:** 10.1038/s41598-026-59505-8

**Published:** 2026-08-09

**Authors:** Merle Retzlaff, Hannes Hollborn, Maisa Omara, Oliver Schierz

**Affiliations:** 1 Department of Prosthetic Dentistry and Materials Science, Medical University of Rostock, Rostock, Germany; 2 Clinic and Polyclinic for Ear, Nose and Throat Medicine, Head and Neck Surgery ‘Otto Körner’, Medical University of Rostock, Rostock, Germany

**Keywords:** Artificial intelligence, Dentistry, Digital dentistry, Technology adoption, Dental practice, Cross-sectional study, Business and industry, Health care, Mathematics and computing

## Abstract

Administrative tasks substantially limit the time available for direct patient care in dentistry. The use of artificial intelligence (AI) can reduce administrative workload and improve practice efficiency, however its integration into routine dental practice remains limited. Existing research often focuses on specific AI applications or surveying digitally proficient practitioners, leaving practices with lower levels of digitalization underrepresented. This exploratory study establishes a baseline assessment of digitalization and AI readiness in Mecklenburg-Western Pomerania, Germany. German dental practices with varying degrees of digital maturity were included to assess existing digital infrastructure, current and intended AI use, and perceived barriers to adoption. A questionnaire-based survey was conducted among all dental practice owners in the German federal state of Mecklenburg–Western Pomerania. The exploratory survey assessed digital infrastructure, AI use, and perceived barriers. The data was analysed descriptively. A total of 163 dental practice owners participated, including a substantial proportion from rural regions (55%). Most practices reported exclusively digital patient records and appointment management, while the use of advanced digital tools varied. A minority had already implemented AI applications, which included support for administrative and diagnostic tasks, documentation, and radiographic evaluation. The most frequent reported barriers to AI adoption were perceived additional costs, concerns about increased workload, and limited familiarity with AI applications. AI adoption in dental practices in a low-density region remains limited, with practitioners favouring tools that reduce administrative workload. While digital infrastructure is increasingly available, barriers such as financial risk, implementation effort, and limited familiarity persist.

**Supplementary Information:**

The online version contains supplementary material available at 10.1038/s41598-026-59505-8.

## Introduction

Physicians currently spend up to four hours per working day on administrative tasks^1^. This substantial administrative workload significantly reduces the time available for direct patient care. The use of AI can alleviate administrative burden, allowing dental clinicians to devote more time to direct patient care. Within healthcare administration, AI technologies can predict waiting times during open consultation hours^2^, support with inventory management^3^, or enhancing document workflows within the hospital organisation^4^. In the field of patient treatments, AI can support histopathological analysis of tissue sections^5^ or facilitate faster analysis of CT scans in acute stroke diagnostics^6^.

Beyond these medical applications, AI tools have also been introduced across multiple dental disciplines, such as in paediatrics, with the ability to identify primary proximal caries lesions on bitewing radiographs^7^. Furthermore, AI can be used in the field of aesthetic dentistry for customised smile design^8^, in orthodontics for landmark detection^9^ or for automated age prediction in forensic dentistry^10^. Surveys among dental students show largely positive attitudes toward artificial intelligence, with 72–80% anticipating that AI will substantially advance or transform dental practice and 64–65% supporting AI-based diagnostic systems^11,12^. At the same time, nearly half report ethical concerns, and about 50% reject the idea that AI could replace dentists^11,12^. The impact of digital technology on healthcare delivery is expected to increase substantially by 2030^13^.

Despite the rapidly expanding range of artificial intelligence applications with the potential to improve clinical decision-making, patient outcomes, and practice efficiency, the integration of AI into routine medical and dental practice (DP) remains limited^14,15^. Previous studies have primarily focused on specific AI applications or assessed practitioners’ attitudes through predominantly web-based surveys^16–19^. These designs may inherently favour digitally engaged and urban-based participants^20^. Consequently, DPs with lower levels of digitalization, particularly those in rural settings, are underrepresented, limiting the generalizability of current findings^20,21^.

The successful implementation of AI in healthcare is fundamentally dependent on the availability of adequate digital infrastructure^19^. However, digital readiness cannot be assumed across all DPs, as demonstrated by the challenges associated with the digital health initiative in Germany to implement the electronic health record nationwide^22^. The absence of a systematic assessment of the current level of digitalization in dental practices represents an insufficiently explored area in the current literature. It remains unclear to what extent DPs are technically prepared for AI adoption, which digital tools are already in use, and what barriers impede further implementation - particularly among practitioners with limited technological affinity or research engagement.

To address this gap, the present exploratory study aims to establish a baseline assessment of digitalization and AI readiness among German DPs using a questionnaire-based approach. By including both digitally proficient and primarily analogue practices from urban and rural areas, the study examines existing digital infrastructure in Mecklenburg-Western Pomerania, current and intended AI use, and perceived barriers to adoption. Given the structural characteristics of the region, limitations in digital infrastructure may represent a relevant barrier to AI uptake. The findings are intended to provide an insight into the practical needs and realities of everyday dental care in Mecklenburg-Western Pomerania, Germany.

## Methods

The study protocol was carried out in accordance with the Declaration of Helsinki^23^ and was approved by the Ethics Committee of the University Medicine Rostock, Germany (Register number: A 2025 − 0112).

### Questionnaire development

We firstly conducted a brief literature review with PubMed and Google Scholar to identify the items to be included into the questionnaire. Based on this information, an initial pilot questionnaire was then developed and evaluated for clarity and relevance. The target group comprised owners of DPs in the federal state of Mecklenburg-Western Pomerania, Germany.

**Fig. 1:**
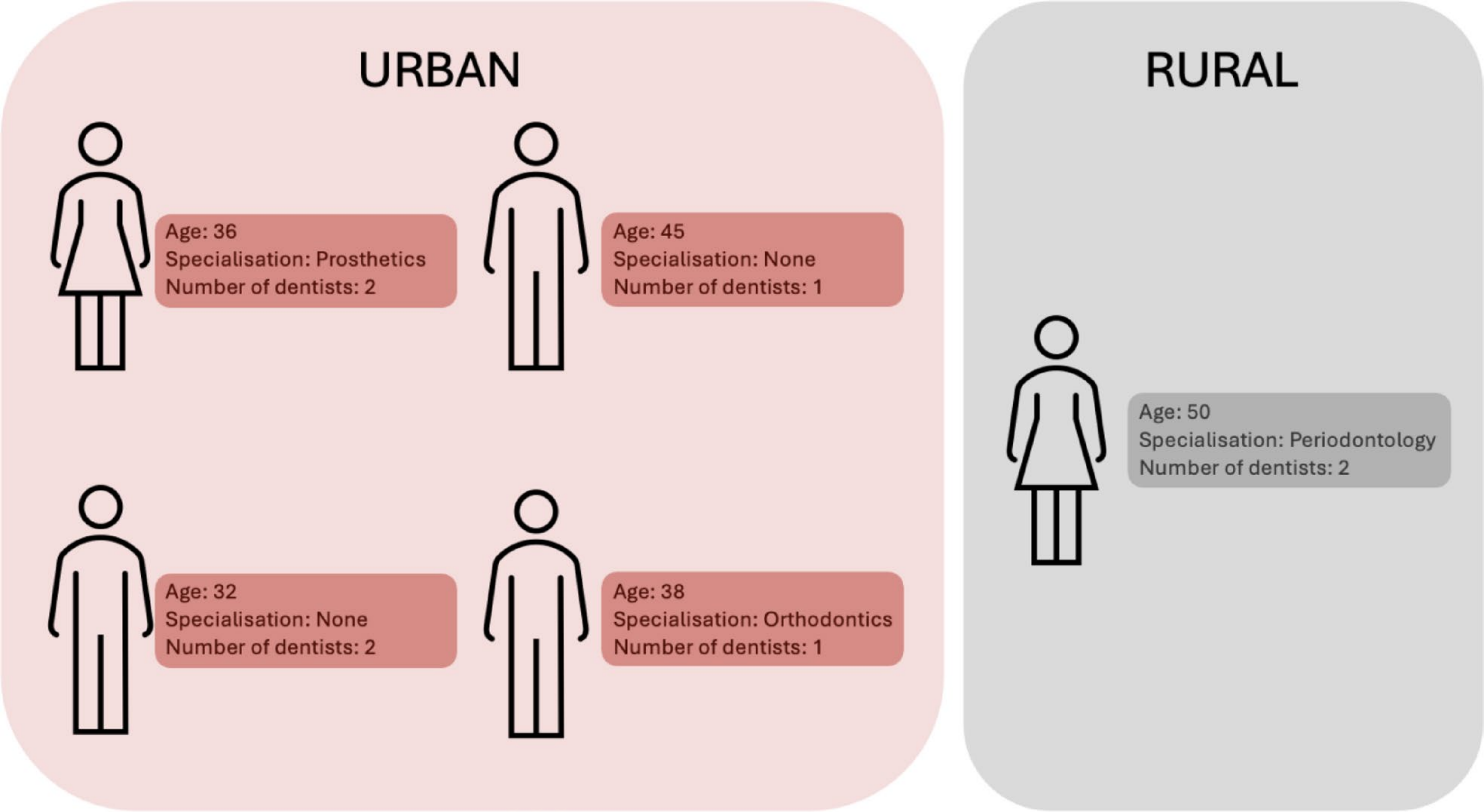
Profile of the DP owners who participated in the pilot questionnaire testing.

The questionnaire covers aspects such as demographic and professional characteristics (e.g., postal code, gender, age, specialization), and assesses the participants towards perceptions, attitudes, and expectations regarding AI. As all responses were self-reported and not externally verified, the assessment of knowledge and attitudes should be considered subjective overall. The questionnaire consists of three parts. The first part collected general data about the DP to characterize the study sample. These include owner’s age and gender, the postal code, the number of employees, and possible specializations of the DP. The second part aimed to gather information on the general practice organization to evaluate the status of digitalization in the DP. Those include the main means of communication between practice and patients, the software systems used for documentation and appointment management, and the digital applications being used regularly in the DP. The third and final part focused on the knowledge and use of AI. The aim was to remain within a two-page A4 limit to increase the response rate. To evaluate the clarity and comprehensibility of the questionnaire and to identify potential ambiguities or misinterpretations a pretest was conducted. The pilot questionnaire was revised through direct communication with five selected DP owners using the think-aloud method. Figure 1 shows the different profiles of the DP owners selected for the piloting phase. The questionnaire was specifically developed for this study and, although pretested for clarity, has not undergone formal validation. The selected DPs represented different age groups, genders, and specializations. All participants were responsible for managing their own dental practices. The final version of the questionnaire was then created using the digital EvaSys survey tool (Version V9.1, EvaSys GmbH, Lüneburg, Germany) to conduct a hybrid survey.

### Questionnaire distribution

A general QR code linking to the survey was published in the June 2025 issue of the Dental Association of Mecklenburg-Western Pomerania newsletter, which resulted in seven responses recorded in EvaSys. To maximise the response rate, the questionnaire was additionally distributed to DPs by postal mail in a pseudonymised paper form. The contact details of the dental practice owners were obtained from the official database of the Dental Association of Mecklenburg-Western Pomerania (https://zaekmv.de/zahnarztsuche), which, as of March 17th, 2025, listed 778 registered dental practices in the federal state. Dental practices were located in urban areas (*n* = 354), and in rural areas (*n* = 424). The mailed study package consisted of a cover letter with general information about the study, a participant information sheet and the two-page questionnaire. In order to target both digitally proficient and predominantly analogue practices, this survey could be completed either digitally via a unique QR code or on paper. Participation was voluntary and responses could be submitted via e-mail, facsimile, or direct entry into the EvaSys online platform. By returning the questionnaire, the participants provided their consent, as stated in the heading of the questionnaire (see supplementary file “Questionnaire-English”). The university mailroom dispatched the letters on June 12th, 2025, and 773 letters were successfully delivered. Five of these letters could not be delivered. On July 10th, 2025, 293 reminder e-mails were sent to all dental practices for which an e-mail address was available in the Mecklenburg-Western Pomerania Dental Association database. Of these e-mails, 15 could not be delivered.

### Questionnaire analysis

After a 15-week waiting period, the overall response rate was 21%, with 163 completed questionnaires received, all of which were included in the analysis. Of these, 148 were obtained in direct response to the postal mail-out, 36 were returned via e-mail, 13 via facsimile, 46 via postal mail, and 53 were completed directly on the online platform. An additional six responses were received following the publication in the professional journal, and nine further responses were obtained in reaction to the e-mail reminder.

This study was designed as an exploratory, descriptive cross-sectional survey; therefore, no formal sample size calculation was performed, and data analysis was descriptive. Open-ended questions were manually coded and categorised by the first author. To improve consistency, the identified categories were discussed with the co-authors during the analysis process. As the qualitative component of this exploratory study was descriptive in nature, no independent second coding or formal inter-rater reliability assessment was performed. Incomplete questionnaires were also included. Response numbers therefore vary between survey items. Microsoft Excel (Version 16.104, Microsoft, 2026) was used for data management and analysis.

## Results

### First section: general data

**Fig. 2:**
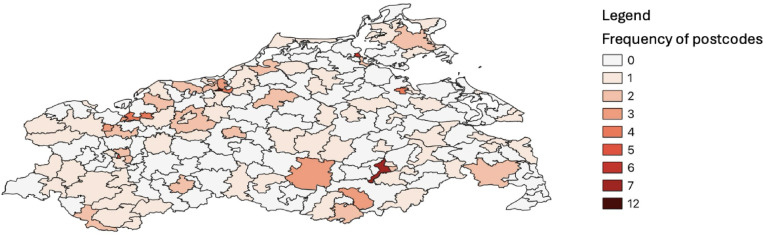
Topographical distribution of the participating dental practices across Mecklenburg- Western Pomerania, Germany.

Out of 163 responding dental practices in this study 79 were exclusively female-owned. The mean age was 51.1 years. The majority (*n* = 90) were situated in the rural part of Mecklenburg- Western Pomerania, Germany. The topographical distribution of the participants is shown in Fig. 2. The majority (*n* = 124) were non-specialised dentists. Most participating dental practices were owned by one or two dentists. Of the participants, 117 don’t have a social media presence. The detailed figures are presented in Table 1.

**Table 1:** Results from part 1 of the questionnaire (general data) demographic characteristics and baseline information of the study participants.

Category	Characteristic	Frequency *n* (Percentage)
Gender	Female	79 (50)
	Male	69 (43)
	Mixed	11(7)
	n_Total_	158 (100)
Location of the dental practice (postal code)	Urban (defined as a city > 40.000 inhabitants)	73 (45)
	Rural	90 (55)
	nTotal	160 (100)
Age	20–29 years	1 (1)
	30–39 years	23 (15)
	40–49 years	52 (33)
	50–59 years	44 (28)
	60–65 years	26 (16)
	> 65years	14 (9)
	nTotal	160 (100)
Specialisation (multiple answers possible)	None	124 (76)
	Prosthetics	16 (10)
	Orthodontics	16 (10)
	Oral surgery	11 (7)
	Paediatric dentistry	10 (6)
	Endodontics	10 (6)
	Periodontology	8 (5)
	nTotal	163 (100)
Social Media Presence	Yes	45 (27)
	No	117 (73)
	nTotal	162 (100)

Category	Characteristic	Average (Range Min–Max)
Number of Employees (Average)	Dentist	1.57 (1–6)
	Dental assistant	4.67 (1–17)
	Trainees	0.64 (0–9)
	nTotal	162 (100)

### Second section: practice organisation

For questionnaire distribution, 115 practices use paper forms, while 43 use tablets. Regarding patient management, 104 practices rely solely on digital patient files and calendars, whereas 19 remain fully analogue. As AI applications typically require a digital infrastructure, we also assessed the use of additional digital tools: 147 practices already employ digital X-rays and 57 use intraoral scanners. The complete set of data is shown in Table 2.

**Table 2:** Results from part 2 of the questionnaire (Practice Organisation) analysis of the digital infrastructure.

Category	Characteristic	Frequency *n* (Percentage)
Distribution of questionnaires (multiple answers possible)	Paper-based	115 (56)
	Tablet	43 (21)
	QR-code	12 (6)
	E-mail	12 (6)
	Website	11 (6)
	Other: laminated paper	6 (4)
	Other: tool (e.g., app)	3 (2)
	nTotal	163 (100)
Digitalisation of the patient file and calendar	Exclusively digital	104 (64)
	Exclusively analogue	19 (12)
	Both	40 (25)
	nTotal	163 (100)
Digital applications used (multiple answers possible)	None	13 (8)
	Digital X-ray	147 (90)
	Intraoral scanners	57 (35)
	AI-based X-ray	15 (9)
	Digital facebow	7 (4)
	Other: hygiene management	8 (5)
	Other: 3D-printing, Cerec	6 (4)
	nTotal	163 (100)

### Third section: AI usage

Among participants, 114 were aware of AI applications in dentistry and 109 had no prior contact with AI in their practice. Tasks perceived as inefficient or time-consuming included documentation (*n* = 52), accounting (e.g., cost estimates) (*n* = 29), patient information (*n* = 21), and appointment management (*n* = 21) - with respondents allowed to select multiple options.

When asked which tasks they could imagine delegating to an AI, 45 considered documentation, 36 X-ray evaluationand, 27 appointment management suitable, while ten participants would not delegate any tasks. Conversely, participants were generally reluctant to delegate tasks involving direct patient contact, such as patient information (*n* = 27), treatment (*n* = 26), and appointment management (e.g. at the reception or telephone) (*n* = 23), whereas eight indicated no restrictions.

Regarding barriers to AI implementation, respondents could select multiple reasons. The main factors were additional costs (*n* = 91) and fear of additional workload (e.g., maintenance) (*n* = 68). The numerical data is set out in full in Table 3.

**Table 3:** Results from part 3 of the questionnaire (AI Usage) specific questions on AI awareness, desired tasks, and barriers to implementation.

Category	Characteristic	Frequency *n* (Percentage)
Awareness of AI in dentistry	Yes	114 (72)
	No	45 (28)
	nTotal	159 (100)
Contact with AI in the dental practice	Yes	109 (68)
	No	52 (32)
	nTotal	161 (100)
Tasks being perceived as inefficient (multiple answers possible)	Documentation	52 (40)
	Accounting	29 (22)
	Quality management	21 (16)
	Patient information	21 (16)
	Appointment management	21(16)
	Hygiene	17 (13)
	Bureaucracy/administration	13 (10)
	Human resource management	8 (6)
	X-ray evaluation	7 (5)
	Other	23 (18)
	nTotal	130 (100)
Tasks wished to be performed by AI (multiple answers possible)	Documentation	45 (36)
	X-ray evaluation	36 (29)
	Appointment management	27 (21)
	Accounting	21 (17)
	Patient information	16 (13)
	None	10 (8)
	Quality management	7 (6)
	Hygiene	7 (6)
	Stock material order	7 (6)
	Bureaucracy/administration	4 (3)
	Human resource management	5 (4)
	Other	21(17)
	nTotal	126 (100)
Tasks not wished to be performed by AI (multiple answers possible)	Doctor-patient communication	27 (29)
	Treatment	26 (28)
	Appointment management (telephone, reception)	23 (25)
	Contact with patient	11 (12)
	None	8 (9)
	Patient file	6 (7)
	X-ray	4 (4)
	Documentation	3 (3)
	Other	9 (10)
	nTotal	92 (100)
Barriers to AI implementation	Additional cost	91 (57)
	Afraid of additional workload (e.g., maintenance)	68 (43)
	No contact yet with AI	59 (37)
	Immature technology	57 (36)
	Lack of fitting appliances	53 (33)
	Data security issues	45 (28)
	No trust in the technology	21 (13)
	Other: imminent retirement	6 (4)
	Other: no necessity	5 (3)
	Other: in the process of implementation	2 (1)
	nTotal	159 (100)

## Discussion

This exploratory study provides a baseline assessment of digitalization and AI readiness in dental practices in Mecklenburg-Western Pomerania. By offering multiple response channels to the participants the study deliberately included less digitally affine practices that are typically underrepresented in online-only surveys^16,20^. This approach enables a more realistic evaluation of real-world adoption conditions in routine dental care, than online-only surveys typically allow.

### First section: general data

The participating practices broadly reflected the demographic and geographic characteristics of a low population-density federal state^24^ with a balanced distribution between rural and urban areas. The age distribution of practice owners (see Fig. 2) was comparable to official data from the general population in Mecklenburg-Western Pommerania^25^ suggesting that the sample broadly reflected the regional demographic structure. However, given the descriptive design, these observations cannot be interpreted as causal relationships. The limited social media presence and the high proportion of non-specialized practices may also reflect regional practice characteristics that could influence digital workflows, although this cannot be confirmed within the scope of this study. Balanced urban–rural sampling is particularly relevant, as previous studies have reported lower adoption rates of digital technologies and AI in rural healthcare settings, partly due to economic and infrastructural constraints^26,27^. Tajirian and Binstock^28^ describe fragmented and uneven AI implementation across Canadian healthcare, with workflow-embedded tools showing stronger adoption while community and remote settings remain underrepresented. A previous study by Maita et al.^29^ suggests that integrating digital tools might ensure an equitable access to healthcare infrastructure in rural areas. Rural medical facilities are reportedly under significant pressure and short of staff, so it is crucial that they can maximise the efficiency and quality improvements that AI tools could offer^30^.

### Second section: practice organisation

Most practices reported having basic digital infrastructure indicating that foundational prerequisites for AI implementation are widely available across Mecklenburg-Western Pomerania, Germany. That is consistent with a study by Schnitzler et al.^31^ where 85% of the participants are reported to use the digital patient file in Germany. At the same time, the adoption of more advanced digital tools (intraoral scanner 35%) varied considerably, suggesting heterogeneous levels of digital maturity. These findings are comparable with a study by Schlenz et al.^18^, who reported intraoral scanners use by one third of their participants. Research has also shown that age and clinic size influence the use of digital technologies^13^. Previous studies suggest that the adoption of digital technologies varies across dental specialties, with reported digital workflow implementation rates of 22% among oral and maxillofacial surgeons, compared with 44% among general practitioners and 45% among orthodontists^32^. Evidence from other healthcare settings further suggests that partial or poorly integrated digitalization can limit efficiency gains and reduce the potential benefits of advanced technologies^33,34^.

### Third section: AI usage

Economic considerations emerged as the most frequently reported barriers to AI adoption. These findings may indicate that reluctance toward AI is associated with perceived financial risk, implementation effort, and uncertainty about technological maturity rather than fundamental scepticism. Similar findings have been reported with previous studies identifying financial concerns, workflow misalignment, and readiness of AI tools as key barriers to adoption in healthcare^35,36^. Our results regarding general knowledge among dentists (72%) are consistent with Pringle et al.‘s findings of 70%^37^, whereas Bedia et al. and Grosu et al. have reported a prevalence between 56–60%^38,39^.

Practitioners predominantly expressed interest in AI support for documentation (36%) and administrative tasks (ranging between 17 and 29%) which are both associated with high time expenditure and limited direct patient value. In contrast, AI involvement in the direct clinical workflow and patient interaction were largely rejected. Similar task-specific acceptance patterns have been previously reported, underscore the thesis that AI is most readily accepted when it supports efficiency without compromising clinical autonomy or direct patient interaction, whereas tools closer to clinical decision-making are met with more caution^39,40^. While some studies suggest greater comfort with AI in efficiency‑oriented tasks, other work shows very high acceptance of AI that directly supports diagnosis and treatment planning^41–43^. Furthermore, a narrative review of AI in dentistry highlights the infrastructural, ethical, and integration challenges that must be addressed before it can be implemented more widely in clinical practice^44,45^.

Notably, several perceived barriers, particularly concerns regarding technological maturity and the availability of suitable tools, may indicate limited awareness of currently available AI applications rather than actual technical shortcomings. Many of the AI applications identified as desirable by respondents are already commercially available^46–48^, in which the clinically mature AI applications are largely confined to diagnostics, documentation, and image analysis, while more complex clinical and patient-facing uses remain limited^49^. Limited information regarding available AI applications may contribute to perceived financial risk and hesitancy toward adoption, emphasizing education and transparent communication as key economic enablers alongside financial incentives^35,50^. The flowchart in Fig. 4 illustrates key barriers and enabling factors affecting the integration of artificial intelligence into routine dental practice.

Overall, the findings of this study suggest that AI adoption in dental practice may be influenced less by technical feasibility and more by economic risk perception, implementation effort, and heterogeneous levels of digital maturity. While basic digital prerequisites are often met, targeted measures (e.g. clear, resourced digital strategy^51^, educational reforms^31^ may support the translation of existing infrastructure into economically viable and sustainable AI implementation. Integrating targeted AI content into dental curricula may support smoother implementation in practice, given that many dental students describe limited knowledge and express a desire for more structured AI teaching^52,53^.

A strength of this study is its hybrid data collection approach, which reduces digital selection bias and enables participation from practices with varying levels of digital affinity. The inclusion of a substantial proportion of rural practices provides exploratory insight into a setting that is often underrepresented in digitally focused studies^16–19^.

We also want to discuss the following limitations of our findings. Firstly, no subgroup analyses comparing rural and urban practices were conducted, which limits conclusions regarding potential differences between these settings. Secondly, the questionnaire was not formally validated, and no statistical validation (e.g., assessment of internal consistency) was performed. Consequently, the findings should be interpreted with caution, particularly regarding the generalizability and comparability of the results with other studies. The descriptive nature of the analysis limits the ability to draw conclusions beyond the observed response patterns and may affect comparability with other studies. Thirdly, the qualitative analysis was based on manual single-author coding without independent validation or formal inter-rater reliability assessment. Although the identified categories were discussed with the co-authors, subjective interpretation and limited reproducibility cannot be excluded. Finally, the study was geographically limited to Mecklenburg-Western Pomerania, which may restrict the generalizability of the findings to other regions. The response rate of 21% is comparable to similar postal surveys but does not ensure representativeness. Consequently, the findings should be interpreted primarily as an exploratory regional assessment and may not be directly generalizable to all dental practice settings. The descriptive design precludes causal inference, and all data rely on self-report, which may introduce recall or social desirability bias.

The findings highlight several implications for policy and practice with targeted funding, transparent information, and practice-oriented education emerging as key enablers for AI adoption in our cross-sectional study. These enablers are consistent with prior research on healthcare AI implementation in healthcare, which emphasizes funding, education, implementation capacity, and supportive policy environments as important enabling conditions^54^.

Future research should include multi-regional samples and perform subgroup analyses to explore potential differences between practice types. Mixed-methods approaches combining quantitative surveys with qualitative interviews could provide deeper insights into adoption barriers and facilitators. Replication studies may benefit from hybrid survey strategies to minimize digital selection bias.

## Conclusion

This exploratory study provides insight into patterns of AI adoption in dental practices in an area with a low population density, highlighting both structural and economic factors that influence uptake. Digital infrastructure is seemingly becoming more accessible across DPs in Mecklenburg- Western Pomerania, Germany, forming an essential foundation for the implementation of AI. However, the adoption of AI applications remains selective, with practitioners favouring tools that reduce their administrative workload rather than those that affect clinical decision-making or patient interaction. Barriers such as perceived financial risk, implementation effort, and limited familiarity with AI persist. Overall, supporting practitioners with education, practical resources, and financial or organizational support may be relevant to consider in order to realize the full potential of AI in dental care.

**Fig. 3:**
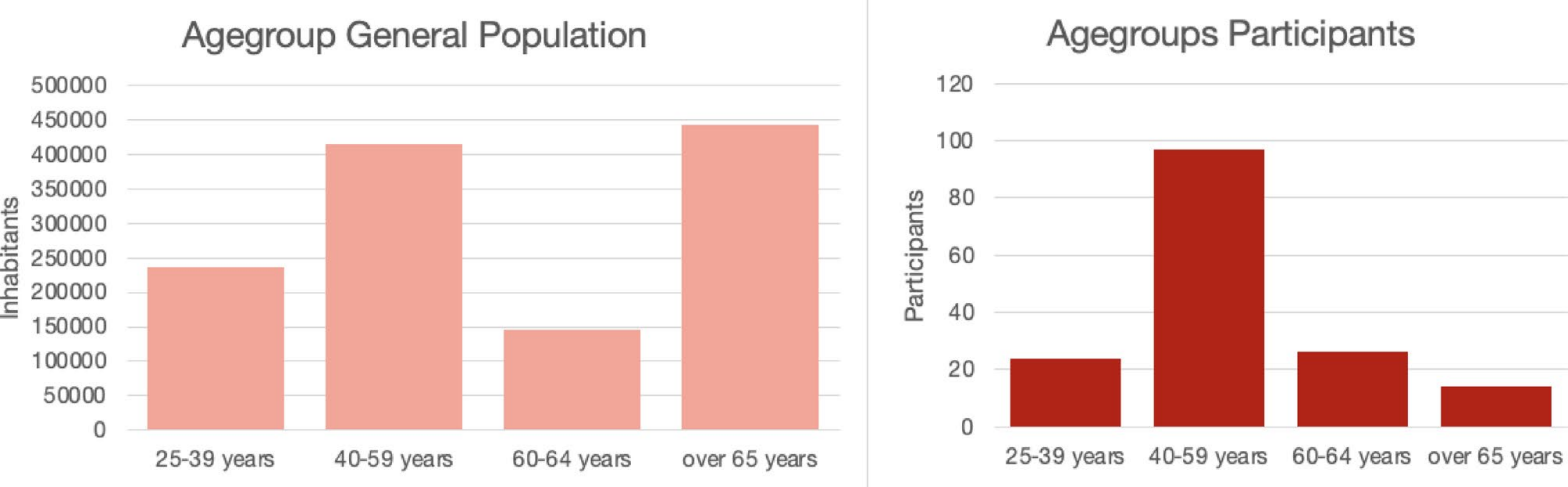
Age distribution of study participants in comparison with the general population. in Mecklenburg-Western Pomerania, Germany^25^.

**Fig. 4:**
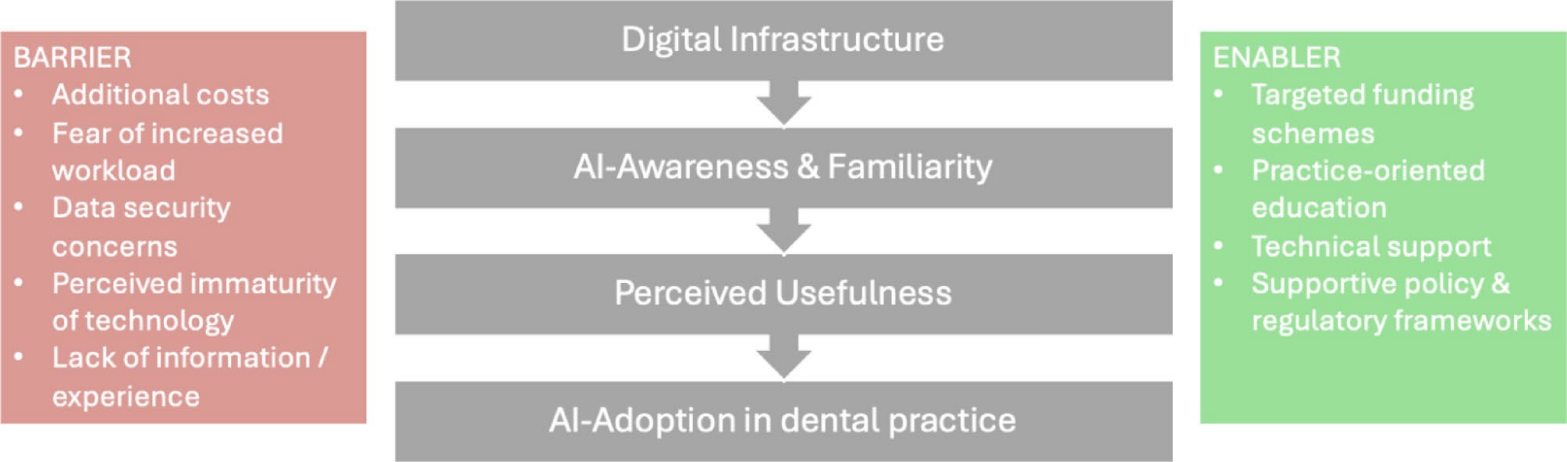
Barriers and enablers influencing AI adoption in dental practices.

## Supplementary Information

Below is the link to the electronic supplementary material.

Supplementary Material 1

Supplementary Material 2

## Data Availability

The datasets generated during the present study are available from the corresponding author upon reasonable request.
