# Supplementary material for: An exploratory survey of barriers and enablers of AI adoption in rural and urban dental practices in Mecklenburg-Western Pomerania, Germany
Source: Sci Rep. 2026 Aug 9;16:24705. doi: 10.1038/s41598-026-59505-8 (PMC13458717; doi:10.1038/s41598-026-59505-8)
Supplement: Supplementary file 1 [file 41598_2026_59505_MOESM1_ESM.pdf]

MUSTER

Poliklinik für zahnärztliche Prothetik und Werkstoffkunde, Universitätsmedizin Rostock
ZÄ Merle Retzlaff

Mit Übermittlung der Informationen willige ich deren Datenverarbeitung ein

Bitte so markieren: ☐ ☒ ☐ ☐ ☐ Bitte verwenden Sie einen Kugelschreiber oder nicht zu starken Filzstift. Dieser Fragebogen wird maschinell erfasst.
Korrektur: ☐ ■ ☐ ☒ ☐ Bitte beachten Sie im Interesse einer optimalen Datenerfassung die links gegebenen Hinweise beim Ausfüllen.

1. Abschnitt: Randdaten zur Praxis

1.1 Postleitzahl der Praxis

1.2 Alter der Praxisinhaber:innen (Bei Mehreren: zählen Sie bitte auf)

1.3 Geschlecht der Praxisinhaber:innen
- ☐ Weiblich
- ☐ Männlich
- ☐ Divers
- ☐ Gemischt

1.4 Spezialisierung der Praxis
- ☐ Keine
- ☐ Kieferorthopädie
- ☐ Oralchirurgie
- ☐ Kinderzahnheilkunde
- ☐ Parodontologie
- ☐ Endodontologie

1.5 Mitarbeiterzahl (Bitte auf volle Stellen addieren)
Zahnärzt:innen / Helfer:innen / Azubis

__ __ / __ __ / __ __

2. Allgemeine Praxisorganisation

2.1 Auf welchem Weg kontaktieren Patient:innen häufig Ihre Praxis? (Mehrfachantwort möglich)
- ☐ Telefonanruf
- ☐ SMS
- ☐ Email
- ☐ Website
- ☐ Andere

2.2 Andere

2.3 Auf welchem Weg nehmen Sie häufig Kontakt zu Patient:innen auf? (Mehrfachantwort möglich)
- ☐ Telefonanruf
- ☐ SMS
- ☐ E-Mail
- ☐ Website
- ☐ Andere

2.4 Andere

2.5 Schätzen Sie: Wie oft wird die Rezeption durchschnittlich am Tag kontaktiert? Kreuzen Sie an

0 ☐☐☐☐☐☐☐☐☐☐☐ 100

2.6 Welche sind die häufigsten Gründe für eine Kontaktaufnahme durch Patient:innen? (Mehrfachantwort möglich)
- ☐ Terminabsagen- und Änderungen
- ☐ Terminneuvereinbarung bei (akuten) Beschwerden
- ☐ Terminneuvereinbarung bei Behandler:innen Wechsel
- ☐ Termin für Kontrolluntersuchungen
- ☐ Andere

2.7 Andere

2.8 Auf welche Weise werden Fragebögen in Ihrer Praxis ausgegeben?
- ☐ Papier
- ☐ Gar nicht
- ☐ E-Mail
- ☐ Website
- ☐ Tablet in Praxis
- ☐ QR-Code
- ☐ Andere

MUSTER

2. Allgemeine Praxisorganisation [Fortsetzung]

2.9 Andere

2.10 Welche Praxissoftware wird aktuell verwendet? (Mehrfachantwort möglich)

- ☐ Terminverwaltung und Patient:innenakte sind in derselben Software
- ☐ Wir arbeiten derzeit mit einem analogen Terminkalender
- ☐ Wir arbeiten derzeit mit einer analogen Patient:innenakte

2.11 Verwendete Praxissoftware für Terminkalender

2.12 Verwendete Praxissoftware für die Patient:innenakte

2.13 Werden darüber hinaus noch weitere digitale Anwendungen in der Praxis benutzt?

- ☐ Nein
- ☐ Intraoralscanner
- ☐ digitaler Gesichtsbogen
- ☐ digitales Röntgen
- ☐ KI-basierte Röntgenauswertung
- ☐ Andere

2.14 Andere

3. Spezielle Fragen zu künstlicher Intelligenz (KI)

3.1 Sind Ihnen Einsatzmöglichkeiten von KI in der Zahnmedizin bekannt?

- ☐ Ja
- ☐ Nein

3.2 Haben Sie bereits im Praxiskontext Kontakt mit Anwendungen künstlicher Intelligenz gehabt?

- ☐ Nein
- ☐ Ja, folgende:

3.3 Ja, folgende:

3.4 Sind Sie auf sozialen Medien vertreten (z.B. Facebook, Instagram…)?

- ☐ Ja
- ☐ Nein

3.5 Welche Abläufe empfinden Sie als besonders zeitaufwendig oder ineffizient in der Praxisorganisation?

3.6 Welche Aufgaben könnten Sie sich vorstellen eine KI ausführen zu lassen?

3.7 Welche Aufgaben/Bereiche würden Sie nicht digitalisieren?

3.8 Warum haben Sie bisher keine KI integriert? (Mehrfachantwort möglich)

- ☐ keine passenden Angebote
- ☐ noch kein Kontakt mit KI-Anwendungen
- ☐ kein Vertrauen in die Technologie
- ☐ Angst vor zusätzlichem Aufwand (z.B. Wartungsaufwand, Bürokratie)
- ☐ Zusätzliche Kosten
- ☐ Datenschutzbedenken
- ☐ unausgereifte Technik
- ☐ Anderes

3.9 Anderes
