# Supplementary material for: An exploratory survey of barriers and enablers of AI adoption in rural and urban dental practices in Mecklenburg-Western Pomerania, Germany
Source: Sci Rep. 2026 Aug 9;16:24705. doi: 10.1038/s41598-026-59505-8 (PMC13458717; doi:10.1038/s41598-026-59505-8)
Supplement: Supplementary file 2 [file 41598_2026_59505_MOESM2_ESM.pdf]

evasys	AI- Applications in the Dental Practice	evasys

Department of Prosthetic Dentistry ZÄ Merle Retzlaff
and Materials Science, Medical University of Rostock
By submitting this information, I consent to its processing

Please mark this way: ☐ ☒ ☐ ☐ ☐ Please use a ballpoint pen or not too thick felt-tip pen. This questionnaire will be read by machine.

Correction: ☐ ■ ☐ ☒ ☐ For optimal data collection, please observe the instructions given on the left when filling in the form.

1. Part: General Data

1.1 Postal code of the dental practice

1.2 Age of the dental practice owner(s) (if several: please list)

1.3 Gender of the dental practice owner
☐ Female ☐ Male ☐ Divers
☐ Mixed

1.4 Specialisation of the dental practice
☐ None ☐ Orthodontics ☐ Prosthodontics
☐ Oral surgery ☐ Pediatric dentistry ☐ Endodontics
☐ Periodontologie

1.5 Number of employees (Please add up to full-time equivalent)
Dentists / Assistants/ Trainees

/ /

2. Part: General Practice Organisation

2.1 By which means do patients frequently contact your practice? (Multiple answers possible)
☐ Phone call ☐ SMS ☐ Email
☐ Website ☐ Other

2.2 Other

2.3 By which means do you frequently contact patients? (Multiple answers possible)
☐ Telefonanruf ☐ SMS ☐ E-Mail
☐ Website ☐ Other

2.4 Other

2.5 Estimate: How often is the reception contacted on average per day? Please tick accordingly.
0 ☐ ☐ ☐ ☐ ☐ ☐ ☐ ☐ ☐ ☐ ☐ 100

2.6 What are the most common reasons for patients to contact you? (Multiple answers possible)
☐ Cancellation or change of appointments ☐ New appointment with (acute) symptoms ☐ New appointment due to change of practitioner
☐ Appointment for check-up examinations ☐ Other

2.7 Other

2.8 How are questionnaires handed out in your practice?
☐ Paper ☐ E-Mail ☐ Website
☐ Tablet in Praxis ☐ QR-Code ☐ Other

MUSTER

2. Part: General Practice Organisation [Fortsetzung]

2.9 Other

[ ]

2.10 Which practice software is currently in use? (Multiple answers possible)

- ☐ Appointment management and patient files are in the same software
- ☐ We currently work with an analogue appointment book
- ☐ We currently work with an analogue patient file

2.11 Practice software used for the appointment management

[ ]

2.12 Practice software used for the patient file

[ ]

2.13 Besides those, are any other digital applications used in the practice?

- ☐ Nein
- ☐ Intraoralscanner
- ☐ digital facebow
- ☐ digitales X-ray
- ☐ AI based X-ray evaluation
- ☐ Other

2.14 Other

[ ]

3. Part: Specific Questions on AI

3.1 Are you familiar with possible applications of AI in dentistry?

- ☐ Yes
- ☐ No

3.2 Have you already had contact with applications of artificial intelligence in the practice context?

- ☐ No
- ☐ Yes, the following:

3.3 Yes, the following:

[ ]

3.4 Are you present on social media (e.g. Facebook, Instagram…)?

- ☐ Yes
- ☐ No

3.5 Which processes in practice organization do you find particularly time-consuming or inefficient?

[ ]

3.6 Which tasks could you imagine being performed by AI?

[ ]

3.7 Which tasks/areas would you rather not digitalize?

[ ]

3.8 Why have you not yet integrated AI? (Multiple answers possible)

- ☐ Fear of additional effort (e.g. maintenance effort, bureaucracy)
- ☐ No contact with AI applications yet
- ☐ No trust in the technology
- ☐ No suitable offers
- ☐ Additional costs
- ☐ Datenschutzbedenken
- ☐ Immature technology
- ☐ Other

3.9 Other

[ ]

MUSTER
